# Density-Dependent Demographic Variation Determines Extinction Rate of Experimental Populations

**DOI:** 10.1371/journal.pbio.0030222

**Published:** 2005-06-07

**Authors:** John M Drake

**Affiliations:** **1**Department of Biological Sciences, University of Notre DameIndianaUnited States of America; Institute of ZoologyUnited Kingdom

## Abstract

Understanding population extinctions is a chief goal of ecological theory. While stochastic theories of population growth are commonly used to forecast extinction, models used for prediction have not been adequately tested with experimental data. In a previously published experiment, variation in available food was experimentally manipulated in 281 laboratory populations of *Daphnia magna* to test hypothesized effects of environmental variation on population persistence. Here, half of those data were used to select and fit a stochastic model of population growth to predict extinctions of populations in the other half. When density-dependent demographic stochasticity was detected and incorporated in simple stochastic models, rates of population extinction were accurately predicted or only slightly biased. However, when density-dependent demographic stochasticity was not accounted for, as is usual when forecasting extinction of threatened and endangered species, predicted extinction rates were severely biased. Thus, an experimental demonstration shows that reliable estimates of extinction risk may be obtained for populations in variable environments if high-quality data are available for model selection and if density-dependent demographic stochasticity is accounted for. These results suggest that further consideration of density-dependent demographic stochasticity is required if predicted extinction rates are to be relied upon for conservation planning.

## Abstract 2

Manipulating the environment of *Daphnia* reveals that extinction risk can be reliably predicted only if density-dependent demographic stochasticity is included and that traditional models ignoring this underestimate the risk of extinction.

## Introduction

As changing global climate and anthropogenic modification of the biosphere increasingly threaten global biodiversity [1, 2], accurately predicting extinctions takes on new urgency [3–5]. Although stochastic models of population growth and decline are commonly used to forecast extinction, quantitative model predictions for population dynamics in randomly fluctuating (i.e., natural) environments have only been poorly supported by experiments [4, 6–7]. In contrast, validation with observational field data [5, 8–10] is beset by numerous difficulties, including low precision [11], low replication [12], small sample size [12, 13], poor data quality [12], and lack of independence [12, 14]. Without empirical validation, viability analyses for threatened and endangered species are subject to doubt, and identifying risk factors for population extinction is difficult. The results reported here support the further use of stochastic models for predicting extinction, but only where detailed information about variation in individual fitness is available for estimating the structure and magnitude of demographic stochasticity.

Two components contribute to random variation in per capita population growth rates: demographic stochasticity and environmental variability [15–18]. Here, demographic stochasticity refers to variation in individual fitness, sampling effects in finite populations, and chance events that affect individuals independently [18–20]. Variation in environmental conditions can cause expected individual fitness to fluctuate, resulting in environmental stochasticity. Defining Δ*N* = *N*_t+1_ − *N*_t_, a model for per capita population growth rate with demographic and environmental stochasticity is



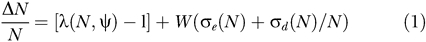



where *λ* is the (possibly density-dependent) expected multiplicative per capita population growth rate depending on population size *N* in the previous time step and parameters *ψ*, *W* is a normal unit variate, and *σ*_e_ *(N)* and *σ*_d_ *(N)* are models for the (possibly density-dependent) effects of environmental and demographic stochasticity, respectively [18]. This is a general model, which can approximate or has as its special cases many common stochastic population growth models, including linear and nonlinear stochastic differential equations [21, 22], birth-death processes [17, 23, 24], and discrete and continuous-time branching process models [25], although it is unclear if these approximations reliably represent the dynamics of small populations [26].

Recent research suggests that demographic stochasticity might have a density-dependent component in the sense that the variance contributed to the population's growth rate by demographic stochasticity, represented by *σ*_d_ in Equation 1, is a function of population size [18, 19, 27]. This phenomenon is independent of the well-known scaling of demographic stochasticity with population size, represented by the ratio *σ*_d_ *(N)*/*N* in the model [15, 16, 18, 25]. While theory predicts that density-dependent demographic variation will occur in all populations in which vital rates are density-dependent [19], and will therefore be ubiquitous in both natural and experimental populations, the severity of density dependence in demographic variation has rarely been investigated, and thus rarely measured [18, 27]. Extinction forecasts that ignore density-dependent demographic stochasticity in populations in which it is present will be biased, possibly severely.

To test for effects of environmental variation on persistence, 281 laboratory populations of water fleas *(Daphnia magna)* were maintained under experimentally controlled conditions for 104 d using three levels of variation in food availability as a source of environmental stochasticity [28]. Here, those data are used to select and test stochastic models of population dynamics. To ensure independence, the data were divided into subsets for model fitting and model testing. Then, as an exploratory analysis, deviations were plotted from expected population growth against population size for populations in the low-variability treatment to suggest how demographic variation might be structured. On the basis of this analysis, a set of models was developed for population growth in fluctuating and constant environments to test for effects of environmental variation. Finally, population growth trajectories with (i) density-dependent demographic stochasticity, and (ii) density-independent demographic stochasticity were simulated to obtain model predicted extinction rates. Simulated extinctions for the model with density-dependent demographic stochasticity were consistent with observed rates of extinction in the test dataset or were only slightly biased, while the model with density-independent demographic stochasticity considerably underestimated observed extinctions. These results suggest that biased estimates of extinction risk resulting from ignoring density-dependent demographic stochasticity may lead to unwarranted optimism concerning the eventual fates of endangered populations.

## Results

### Exploratory Graphical Analysis

After rescaling to isolate density dependence in *σ*_d_ (see Materials and Methods), the scatter plot of observed deviations from expected population growth rate suggested that variation from demographic stochasticity was dependent on population size in populations in the low-variability treatment ( Figure 1). Nonparametric tests for correlation confirmed that the negative relationship between log_e_-transformed deviations and population size was highly significant (Spearman rank-order correlation: ρ = −0.23, *p* < 0.0001; Kendall's τ: ρ = −0.17, *p* < 0.0001; *N* = 342). The average deviation was fit to an exponential model (see Materials and Methods) ( Table 1).

**Figure 1:**
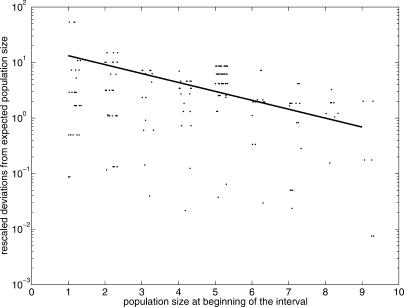
Demographic Stochasticity Is Strongly Density-Dependent in Experimental Populations of *D. magna* Deviations from expected population size were rescaled by multiplying the observed deviation by initial population size for the interval to isolate density dependence in *σ*_d_ (see Materials and Methods). Rescaled deviations are strongly dependent on population size ( *p* < 0.0001). Because observed deviations overlap, obscuring the pattern, points have been jittered in the dimension of the x-axis by the addition of a small amount of random noise.

**Table 1:**
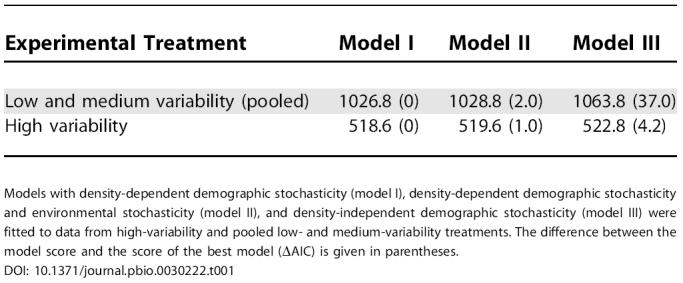
AIC Scores for Ricker Models of Population Growth

### Estimation of Extinction Rates in Experimental Populations

Extinction rates of populations in experimental treatments with low and medium levels of variation were not significantly different, although these were different from the extinction rate of populations subject to a high level of environmental variation (see Materials and Methods). Consequently, observations in the testing dataset from the low and medium variability treatments were pooled for remaining analyses, resulting in a more conservative test of model-predicted extinction rates. The maximum likelihood estimate of extinction rate for populations from the low- and medium-variability treatments in the model-testing dataset was 48.4% (95% confidence interval [CI], 38.0%–58.9%; Figure 2). The maximum likelihood estimate of extinction rate in the high-variability treatment was 70.2% (95% CI, 55.1%–82.7%; Figure 2).

**Figure 2:**
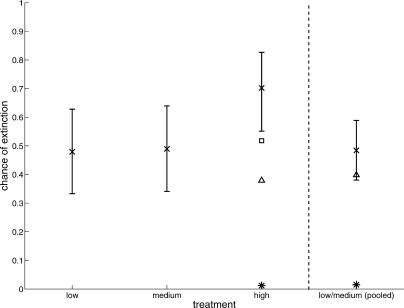
Simple Models Accurately Predict Extinction in Experimental Populations Estimates of the extinction rate in populations of *D. magna* reserved for model testing at three levels of environmental variation were obtained from the likelihood function of the binomial distribution (crosses, 95% CI). Because there was no difference between extinction rates in the low- and medium-variability treatments (see Materials and Methods), data were pooled to obtain a more precise estimate, resulting in a more conservative test (right of dashed line). Model-predicted estimates of extinction rate obtained from models of density-dependent population growth with density-dependent demographic stochasticity fit with independent data (triangles) accurately predicted extinction of populations in the low- and medium-variability treatments, but not the high-variability treatment. The addition of environmental stochasticity (square) improved the prediction, although the chance of obtaining the observed 33 extinctions (or more) out of 47 populations was only 3.3%. The standard model with constant demographic stochasticity (stars) fails to predict extinction in all treatments.

### Model Selection

Model-predicted extinction rates were obtained by fitting a simple Ricker model for population growth, *E*[*λ*] = *λ*_0_ *e^−bN^*, to data in the model-fitting dataset, although a θ-Ricker model was also considered (see Materials and Methods). Moderate to considerable support was obtained for models with only demographic stochasticity compared to models with both demographic and environmental stochasticity, according to Akaike's information criterion (AIC; Table 1). However, there was overwhelming support for a model with density-dependent demographic stochasticity compared to a model with density-independent demographic stochasticity for populations in the low- and medium-variability treatments (ΔAIC = 37.0) and considerable support for populations in the high-variability treatment (ΔAIC = 4.2). These results confirm that density dependence in demographic variance strongly influenced realized population growth in experimental populations.

### Accuracy of Model-Predicted Extinction Rate

The accuracy of model predictions can be assessed by comparing model-predicted extinction rates with the 95% CI for the estimated extinction rate of populations in the model-testing half of the experimental dataset (see Materials and Methods). Overall, models with density-dependent demographic stochasticity accurately predicted population extinction rates within estimable accuracy for populations with low and medium levels of environmental variation, while predictions for populations with a high level of environmental variation were slightly biased ( Figure 2). Models which incorrectly assumed density-independent demographic stochasticity were severely biased and underpredicted observed extinction rates ( Figure 2).

## Discussion

A stochastic Ricker model of population growth with density-dependent demographic stochasticity accurately predicted the chance of extinction, within the power of this experiment to reject the null hypothesis of a difference, or was only slightly biased (Figure 2). Adding environmental stochasticity to the model for populations in the high-variability treatment increased the predicted chance of extinction, consistent with current theory [15–17] and previous experiments [4]. A model in which demographic stochasticity was assumed to be constant failed to predict extinction rates in all experimental treatments, implying that independent data on the structure of individual variation in vital rates, including information about density dependence, is required to reliably forecast extinction.

In this analysis, the accuracy of model predictions was improved by relaxing the usual assumption that demographic stochasticity is density-independent [21, 25]. Since individual fecundity is commonly altered in response to population size or density [29, 30], density-dependent demographic stochasticity resulting from demographic covariation is probably not exceptional in natural populations. Indeed, density-dependent demographic stochasticity will result from any density dependence in vital rates or interactions among individuals that affect demography [19]. Presently, this aspect of population biology is poorly understood (compare [18, 27]). Additionally, the accuracy and precision of these predictions were facilitated by a relatively large, high-quality dataset (*n*_low_ = 342, *n*_med_ = 300, and *n*_high_ = 280). In general, field data will not be so abundant. Thus, an important goal for population biology is to develop methods for obtaining reliable predictions from sparse, low-quality datasets [31].

Planning for increasing threats to rare species from diverse sources, including climate change, resource extraction, habitat modification, and invasive species will require greater and more precise estimates of extinction risk than ever before. While the reliability of theoretical models for predicting extinction in natural ecosystems remains to be established, the results presented here show that accurate predictions of population extinction in variable environments are indeed possible.

## Materials and Methods

### Experiment

Experimental microcosms (*n* = 281) of *D. magna* were maintained on a food resource of *Selanastrum* sp. for 104 d. The daily food availability was experimentally varied at three levels (coefficient of variation = 0, 1, 2), which are referred to as “low,” “medium,” and “high,” respectively, while the long-run average volume of food over time was kept constant across all replicates in all treatments. Extinctions were tabulated and populations were counted daily, although populations with ten or more individuals were simply marked “abundant.” With the exception of these observations, sampling error is negligible. For more detailed methods, see [28]. Prior to this analysis, these data were filtered, retaining only observations from every seventh day for days 1 through 99, corresponding to the approximate generation time, resulting in observations of change in abundance over up to 14 intervals for each population. To achieve independence between data used for model fitting and data used for model testing, populations were assigned to separate datasets. Observations of ten or more individuals were excluded from the dataset for model selection and parameter estimation.

### Exploratory graphical analysis

Because populations in the low-variability treatment were not exposed to any experimentally induced environmental variation, most variation in observed growth rates in these populations should be attributable to demographic stochasticity and is not confounded with environmental stochasticity. Thus, only these data were used for exploratory analysis of density-dependent demographic stochasticity. Estimates of the pairwise multiplicative population growth rate λ^(*N_t_*) between times *t* and *t* + *τ*(*τ* = 7) were obtained from the ratio *N_t_*_+τ_/*N _t_* for observations in the model-fitting dataset from the low-variability treatment. A scatter plot of ^λ^(*N_t_*) versus *N _t_* suggested that density dependence in expected population size is approximately linear on a logarithmic scale (unpublished data). To investigate the structure of deviation from this expectation, data from this treatment were fit to a Ricker model λ^(*N_t_*) = λ_0_*e*^−*bN_t_*^ using ordinary least squares and the squared residuals (*δ _j_* ^2^) were retained. To account for the scaling of demographic variance with population size [15, 16, 18], the residuals were multiplied by the population size at the start of the interval resulting in the rescaled residuals 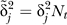. A scatter plot of the rescaled residuals shows clear dependence on population size even after the usual scaling has been accounted for (see Figure 1), which is strongly confirmed by two nonparametric tests for correlation (Spearman rank-order and Kendall's τ). Relatively constant variation around the mean and linear decrease on a logarithmic scale suggest using an exponential function to model variation from demographic stochasticity. Thus, for model selection and estimation, the function *σ*_d_^2^ *(N)* = *e* ^− *αN+β*^ was used. Hyperbolic models of demographic stochasticity were also explored, but these were poorly supported by formal model selection criteria such as AIC, relative to the exponential model.

### Estimating extinction rates in experimental populations

Because populations were independent, each population represents a Bernoulli trial for which the possible outcomes were extinction or persistence. Thus, the chance of extinction for a population in treatment level *i* is binomial with parameter *p _i_*. For *n _i_* populations in the model-testing dataset, of which *x _i_* were observed to go extinct, the maximum likelihood estimate of *p _i_* and 95% CIs on the chance of extinction were determined from the likelihood function for the binomial distribution.

Initially, it was unclear if there was an effect of experimental treatment. Using regression on pooled observations from the entire experimental dataset, Drake and Lodge [28] reported no significant effect of environmental variation on the chance of extinction (*p* = 0.0877). Logistic regression was performed on the testing half of the dataset using treatment as a categorical covariate. The global null hypothesis was rejected by the likelihood ratio test (*p* = 0.0450) but not by Wald's test (*p* = 0.0523), with no coefficients being significantly different than 0 (intercept, *p* = 0.060; β _0_, *p* = 0.174; β _1_, *p* = 0.236). Visual inspection of the data suggests that there might be no difference between the low- and medium-variability treatments (Figure 2), but a difference between these and the high-variability treatment. Repeating logistic regression after pooling observations from the low- and medium-variability treatments unambiguously showed an effect of high variation on the chance of extinction (likelihood ratio test, *p* = 0.013; Wald's test, *p* = 0.015). Therefore, to compare observed extinction rates with model-predicted extinction rates, observations from the low- and medium-variability treatments were pooled for both model fitting and model testing.

### Model selection

The theoretical variance in λ is given by *σ*_e_^2^ *(N)* **+** *σ*_e_^2^ *(N)*/*N,*while the mean can be given by any familiar population dynamical model such as Ricker, θ-Ricker, θ-logistic, or Gompertz growth. Using the exponential model for demographic variation and approximating the unknown distribution of λ with the first two moments, the negative log-likelihood function for the θ-Ricker model of population growth 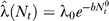 is



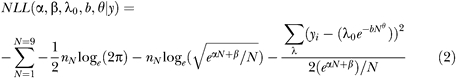



where *λ*_0_ and *θ* can be interpreted as governing the intrinsic rate of increase and the severity of density dependence in the expected growth rate, respectively, and *n _N_* is the number of observed intervals beginning at population size *N*. The negative log-likelihood for this model with the addition of constant environmental stochasticity is



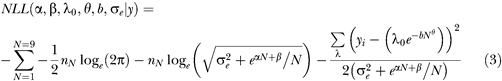



where *σ*_e_ is a parameter representing the average level of variation from environmental stochasticity. Models were fit to data in the model-fitting dataset by minimizing the negative log-likelihood function using the Nelder-Mead simplex. Goodness of fit was quantified using AIC = 2 *NLL*(ψ^|y) + 2 *k* where ψ^ is the vector of maximum likelihood estimates of all parameters, and *k* is the number of parameters estimated. The model with the lowest AIC score is the best fit after accounting for model complexity, while the relative support for a model with the lesser of two AIC scores differing by greater than two is substantial [32].

Overall, the inclusion of the parameter *θ* (which allows for flexibility in the severity of density dependence in expected population growth rate) did little to fit the model and was fixed (*θ* = 1) for the remainder of the analysis, reducing the number of parameters to be estimated by one. AIC scores for remaining models are shown in Table 1. Interestingly, the estimates of *σ*_e_ for data from all experimental treatments were not significantly different from 0, even for the treatment with a high level of experimentally induced variation.

### Accuracy of model-predicted extinction rate

Predicted extinction rates were obtained by simulating 100,000 iterations of the Ricker model with density-dependent demographic stochasticity at maximum likelihood estimates of all parameters for populations in the pooled low- and medium-variability treatments and populations in the high-variability treatment separately using Euler's method [22]. Since predicted extinction rates for populations in the high-variability treatment were biased, and the model without environmental stochasticity was only weakly supported for these populations ( Table 1), 100,000 iterations of the Ricker model were also simulated with density-dependent demographic stochasticity and environmental stochasticity for populations in the high-variability treatment.

The model for stochastic population growth considered here accounts for two factors commonly ignored when predicting the chance of population extinction: density-dependent changes in expected population size and density-dependent demographic stochasticity. Although the effect of density-dependence in *E*[*λ*] on population persistence has been documented [25, 33], the effects of density-dependent demographic stochasticity have not been as extensively studied [18, 27]. Therefore, the chance of extinction that would have been predicted had the incorrect assumption been made that demographic stochasticity was density-independentwas also determined. An estimate of density-independent demographic variance *σ*_d_^2^ *(N)* = *α* was obtained by fitting data from the pooled low- and medium-variability and high-variability treatments to the Ricker model by minimizing the negative log-likelihood function



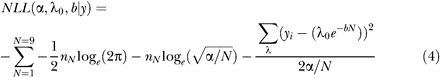



As above, model predicted extinction rates were obtained by simulating 100,000 iterations of the population growth process at maximum likelihood estimates of all parameters.

## Abbreviations

AIC: Akaike's information criterion
CI: confidence interval

## References

[pbio-0030222-b01] Sala OE, Chapin FS, Armesto JJ, Berlow E, Bloomfield J (2000). Global biodiversity scenarios for the year 2100. Science.

[pbio-0030222-b02] McLaughlin JF, Hellmann JJ, Boggs CL, Ehrlich PR (2002). Climate change hastens population extinctions. Proc Natl Acad Sci U S A.

[pbio-0030222-b03] Soulé ME (1987). Viable populations for conservation.

[pbio-0030222-b04] Belovsky GE, Mellison C, Larson C, Van Zandt PA (1999). Experimental studies of extinction dynamics. Science.

[pbio-0030222-b05] Brook BW, O'Grady JJ, Chapman AP, Burgman MA, Akcakaya HR (2000). Predictive accuracy of population viability analysis in conservation biology. Nature.

[pbio-0030222-b06] Forney KA, Gilpin ME (1989). Spatial structure and population extinction—A study with *Drosophila* flies. Conserv Biol.

[pbio-0030222-b07] Belovsky GE, Mellison C, Larson C, Van Zandt PA, Beissing SL, McCullough D (2002). How good are PVA models? Testing their predictions with experimental data on the brine shrimp. Population viability analysis: Assessing models for recovering endangered species.

[pbio-0030222-b08] McCarthy MA, Broome LS (2000). A method for validating stochastic models of population viability: A case study of the mountain pygmy-possum *(Burramys parvus)*. J Anim Ecol.

[pbio-0030222-b09] Ball SJ, Lindenmayer DB, Possingham HP (2003). The predictive accuracy of population viability analysis: A test using data from two small mammal species in a fragmented landscape. Biodiver Conserv.

[pbio-0030222-b10] Lindenmayer DB, Possingham HP, Lacy RC, McCarthy MA, Pope ML (2003). How accurate are population models? Lessons from landscape-scale tests in a fragmented system. Ecol Lett.

[pbio-0030222-b11] Ellner SP, Fieberg J, Ludwig D, Wilcox C (2002). Precision of population viability analysis. Conserv Biol.

[pbio-0030222-b12] Coulson T, Mace GM, Hudson E, Possingham H (2001). The use and abuse of population viability analysis. Trends Ecol Evol.

[pbio-0030222-b13] Fieberg J, Ellner SP (2000). When is it meaningful to estimate an extinction probability?. Ecology.

[pbio-0030222-b14] McCarthy MA, Possingham HP, Day JR, Tyre AJ (2001). Testing the accuracy of population viability analysis. Conserv Biol.

[pbio-0030222-b15] May RM (1973). Stability and complexity in model ecosystems.

[pbio-0030222-b16] Nisbet R, Gurney WSC (1982). Modelling fluctuating populations.

[pbio-0030222-b17] Renshaw E (1991). Modelling biological populations in space and time.

[pbio-0030222-b18] Lande R, Engen S, Sther BE (2003). Stochastic population dynamics in ecology and conservation.

[pbio-0030222-b19] Engen S, Bakke O, Islam A (1998). Demographic and environmental stochasticity—Concepts and definitions. Biometrics.

[pbio-0030222-b20] Kendall BE (1998). Estimating the magnitude of environmental stochasticity in survivorship data. Ecol Appl.

[pbio-0030222-b21] Dennis B, Munholland PL, Scott JM (1991). Estimation of growth and extinction parameters for endangered species. Ecol Monogr.

[pbio-0030222-b22] Allen LSJ (2003). An introduction to stochastic processes with applications to biology.

[pbio-0030222-b23] Bailey NTJ (1964). The elements of stochastic processes with applications to the natural sciences.

[pbio-0030222-b24] Goel NS, Richter-Dyn N (1974). Stochastic models in biology.

[pbio-0030222-b25] Tier C, Hanson FB (1981). Persistence in density dependent stochastic populations. Math Biosci.

[pbio-0030222-b26] Ludwig D (1996). The distribution of population survival times. Am Nat.

[pbio-0030222-b27] Sther BE, Engen S, Islam A, McCleery R, Perrins C (1998). Environmental stochasticity and extinction risk in a population of a small songbird, the great tit. Am Nat.

[pbio-0030222-b28] Drake JM, Lodge DM (2004). Effects of environmental variation on extinction and establishment. Ecol Lett.

[pbio-0030222-b29] Coulson T, Milner-Gulland EJ, Clutton-Brock T (2000). The relative roles of density and climatic variation on population dynamics and fecundity rates in three contrasting ungulate species. Proc R Soc Lond B Biol Sci.

[pbio-0030222-b30] Rödel HG, Bora A, Kaiser J, Kaetzke P, Khaschei M (2004). Density-dependent reproduction in the European rabbit: A consequence of individual response and age-dependent reproductive performance. Oikos.

[pbio-0030222-b31] Holmes EE, Fagan WE (2002). Validating population viability analysis for corrupted data sets. Ecology.

[pbio-0030222-b32] Burnham KP, Anderson DR (2002). Model selection and multimodel inference: A practical information-theoretic approach.

[pbio-0030222-b33] Sabo JL, Holmes EE, Kareiva P (2004). Efficacy of simple viability models in ecological risk assessment: Does density dependence matter?. Ecology.

